# Polarization-dependent fluorescence correlation spectroscopy for studying structural properties of proteins in living cell

**DOI:** 10.1038/srep31091

**Published:** 2016-08-04

**Authors:** Makoto Oura, Johtaro Yamamoto, Hideto Ishikawa, Shintaro Mikuni, Ryousuke Fukushima, Masataka Kinjo

**Affiliations:** 1 Laboratory of Molecular Cell Dynamics, Graduate School of Life Science, Hokkaido University, Sapporo, 001-0021, Japan; 2 Laboratory of Molecular Cell Dynamics, Faculty of Advanced Life Science, Hokkaido University, Sapporo, 001-0021, Japan

## Abstract

Rotational diffusion measurement is predicted as an important method in cell biology because the rotational properties directly reflect molecular interactions and environment in the cell. To prove this concept, polarization-dependent fluorescence correlation spectroscopy (pol-FCS) measurements of purified fluorescent proteins were conducted in viscous solution. With the comparison between the translational and rotational diffusion coefficients obtained from pol-FCS measurements, the hydrodynamic radius of an enhanced green fluorescent protein (EGFP) was estimated as a control measurement. The orientation of oligomer EGFP in living cells was also estimated by pol-FCS and compared with Monte Carlo simulations. The results of this pol-FCS experiment indicate that this method allows an estimation of the molecular orientation using the characteristics of rotational diffusion. Further, it can be applied to analyze the degree of molecular orientation and multimerization or detection of tiny aggregation of aggregate-prone proteins.

Fluorescence correlation spectroscopy (FCS) allows the study of molecular dynamics in solutions and cells^1^,^2^,^3^ while changing the translational diffusion properties of the molecule. Theoretically, according to the Einstein–Stokes equation, rotational diffusion is more sensitive than translational diffusion to changes in the molecular size and interactions. Thus, rotational diffusion measurement is expected to be more sensitive for small changes of molecular size, such as molecular oligomerization and formation of ultrasmall aggregates. Rotational diffusion of fluorophores can be quantified in the FCS using polarized continuous excitation light^4^,^5^. However, owing to several technical limitations, the rotational diffusion of proteins has not been measured using FCS. Previous reports showed that by using pol-FCS, rotational diffusion can be clearly observed in the case of peptide-coated nanorods^6^, Qrod^7^, gold nanorods^8^,^9^,^10^, quantum dots^6^, DNA-DAPI^11^, and large polymers^12^. Further, a positive relationship between translational and rotational diffusion was observed in the viscosity change of a solvent^13^. These studies were performed using molecules that are much larger and brighter than bio-molecules. Other measurement methods of molecular rotational diffusion, such as using nuclear magnetic resonance (NMR)^14^, single molecule method^15^, and time-dependent fluorescence anisotropy decay method^16^, have difficulties of measurement in the case of living cells. The measurement of rotational diffusion in a living cell requires the utilization of at least one fluorescent protein.

Previously, the diffusion of fluorescent proteins has been studied using several methods. For example, fluorescent recovery after photobleaching (FRAP) is a popular method that provides properties of the translational diffusion of fluorescent molecules in cells. However, the FRAP diffusion measurement provides only the effective translational diffusion coefficient under the influence of a barrier structure of surrounding environment, such as cytoskeleton and organelle, because of its relatively wider measurement area of photobleaching. In other words, the FRAP measurement is basically effected by the barriers in the surrounding environment. Thus, it is difficult to estimate the molecular size or formation change using FRAP measurements. To measure the molecular size correctly, rotational diffusion is one of the best approaches. Theoretically, rotational diffusion is not affected by the surrounding barrier structure. In the case of a probe size smaller than the surrounding environment, rotational diffusion is generally much faster than translational diffusion.

Time-resolved fluorescence methods are also powerful tools to study the dynamics and rotational diffusion of fluorescent proteins. However, these methods do not provide translational diffusion properties. In contrast, the pol-FCS method determines both the rotational and translational diffusion coefficients. The distance of translational diffusion and the collisional probability of the molecule are very low in its rotational diffusion relaxation time. Thus, we simultaneously obtain short and long distance spatial information. This means that pol-FCS can simultaneously provide the molecular diffusion properties in nano- and microenvironments using rotational and translational diffusions, respectively. The measured translational diffusion coefficient reflects the microenvironment properties that are defined by the measurement volume. These properties should be the same as in the nanoenvironment as long as the measurement volume is much smaller than the barrier structure. However, some works reported that biomolecules in cells showed anomalous diffusion due to the barrier structure according to FCS measurements. The pol-FCS has the potential to study the diffusional barrier structure in the surrounding environment by comparing the rotational diffusion in nanoenvironment and the translational diffusion in the microenvironment. In this study, rotational and translational diffusions of enhanced green fluorescent proteins (EGFPs) and EGFP tandem-oligomer were measured. We succeeded in the determination of molecular sizes both in phosphate buffered saline (PBS) and also in living cells by using pol-FCS. Furthermore, we found the possibility that the orientation of each EGFP in EGFP tandem-oligomers can be analyzed using pol-FCS.

## Results and Discussion

**Figure 1:**
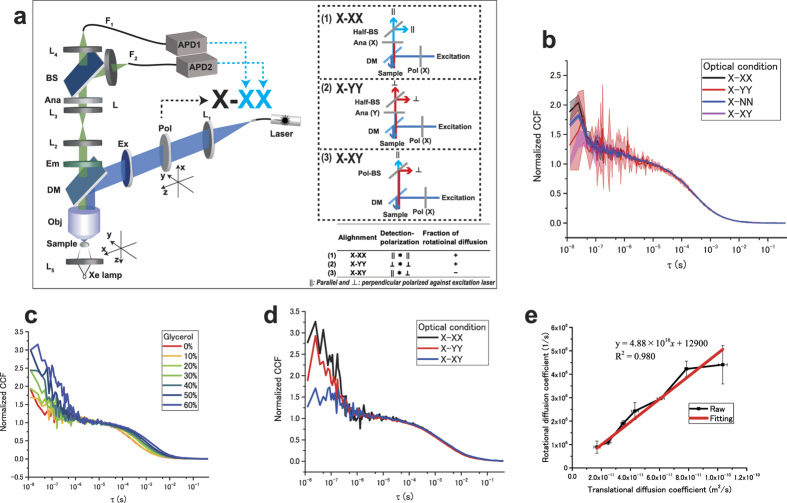
Results of rotational diffusion measurement of EGFPs. (**a**) Schematics of the experimental setup using pol-FCS. L_1_–L_5_: achromatic lenses, Pol: polarizer (polarized plate), Ex: excitation filter, DM: dichroic mirror, Obj: objective lens of a microscope, Em: emission filter, Ana: analyzer, BS: beam splitter, F_1,2_: multimode fibers. Right insets are the schematic optical system in (1) X-XX, (2) X-YY and (3) X-XY. Half-BS: polarization independent 50:50 beam splitter, Pol-BS: polarization dependent beam splitter. Inset table indicates the summary of relationship between the fraction of rotational diffusion and optical polarization. (**b**) The averaged and normalized CCFs of EGFPs under four optical conditions. All condition experiments were performed three times independently. Shaded area means the standard deviation. (**c**) Effect of glycerol concentration on the normalized CCFs of EGFPs in the PBS buffer. (**d**) The typical CCF of EGFPs in 60%/w glycerol. The optical conditions are X-XX, X-NN, and X-XY. (**e**) Relationship between the translational and rotational diffusion coefficients. The red line indicates the linear fitting. Measurements with durations of 30 s were repeated 10 times.

Our newly developed pol-FCS instrument (Fig. 1a) and the global fitting of relaxation time of the triplet state allow the observation of rotational diffusion properties of EGFPs with a clear dependence on solution viscosity. The pol-FCS contains two avalanche photodiodes (APDs) to avoid after-pulses and obtain specific polarization signals (Fig. 1a, Supplementary Note 1, Supplementary Fig. 1). This system was well applied to the living cell system.

### Rotational diffusion measurement of EGFP in solution

First, the pol-FCS measurement was performed to quantify the rotational diffusion time of EGFPs *in vitro*. Using our pol-FCS system, a polarization-dependent component (rotational diffusion) was observed in the time region of 10^−8^ s-10^−7^ s (Fig. 1b) under the X-XX and X-NN optical conditions. X-NN indicates that the excitation laser light is polarized in the X-direction. Thus, “N” means unpolarized: there are no polarization optical elements in the detection side. Alignments (1) X-XX and (2) X-YY (see Fig. 1a, inset table) can be used to reduce after-pulse and obtain the same direction of signals (rotation diffusion fraction positive+). On the other hand, alignment (3) X-XY can be used to reduce after-pulse and measure the opposite-correlated detection of signals (rotation diffusion fraction negative-). As for the X-XX cross-correlation function (CCF), each detector discovered the same rotational tendency of signals from the EGFPs fluorescence. Thus, the fraction of rotational diffusion (*f*_*R*_) is positive (Supplementary Fig. 2). The triplet time (*τ*_T_) of EGFPs was measured to be 1.24 μs using Confocor 3 (Carl Zeiss, Jena, Germany) (Supplementary Fig. 3, Supplementary Note 2). The triplet time was fixed to 1.24 μs in the fitting analysis. Four different optical conditions of the CCF curves were fitted (Supplementary Fig. 4) using the global fitting function of OriginPro 2015 (OriginLab Co., USA), with the same rotational diffusion time that is independent of the optical condition. Supplementary Fig. 4a shows a typical fitting result. The relaxation time of rotational diffusion was calculated as 38 ns in all CCFs using the fitting in Eq. 2. This result is similar to the previous time obtained by time-dependent fluorescence anisotropy decay as 13.8 ± 0.1 ns in a free solution^17^. A difference between this result and previous results may be affected by the different buffer condition. In the case of X-NN, a positive rotational diffusion component was observed as shown in Supplementary Fig 4b. This might be because nonpolarized fluorescence contained a large fraction of *x*-polarized fluorescence and a small fraction of *y*-polarized fluorescence. Regarding X-XY, the fraction of rotational diffusion is small due to a negative rotational correlation between the two signals.

Next, the viscosity dependence of rotational diffusion time of EGFPs was confirmed. The rotational diffusion time of fluorescent molecules is theoretically proportional to the viscosity of a solvent (Equation 7). These experiments were performed independently three times using each sample. The averaged CCFs from the three experiments under the X-XX condition are shown in Fig. 1c. The viscosity-dependent change of rotational diffusion was clearly observed. This result showed that both translational and rotational diffusion times increased with the viscosity of the solvent according to the theory of rotational diffusion^4^,^5^ (Supplementary Fig. 5). While the X-XX case shows a high fraction of rotational diffusion as shown in Fig. 1d, in the X-XY case the amplitude of rotational diffusion is small (Fig. 1d). Using the triplet time fixed nonlinear curve-fitting analysis, the translational and rotational diffusion coefficients were obtained using Eqs 5 and 6 to estimate the hydrodynamic radius (*r*) of the EGFP from pol-FCS results^5^. Figure 1e shows the rotational diffusion coefficient as a function of the translational diffusion coefficient in varying viscosity (Fig. 1c). Using linear fitting analysis, the slope *D*_*R*_*/D*_*T*_ was obtained as 4.88 × 10^16^ m^−2^. Substituting this slope, the hydrodynamic radius was calculated as 3.9 nm (Equation 10). This indicates that EGFPs diffuse in a way that their molecular sizes are bigger than their real size, but are consistent with the results of measurements performed using X-ray crystallography^18^.

### Rotational diffusion measurement of EGFP oligomer in solution and cell

**Figure 2:**
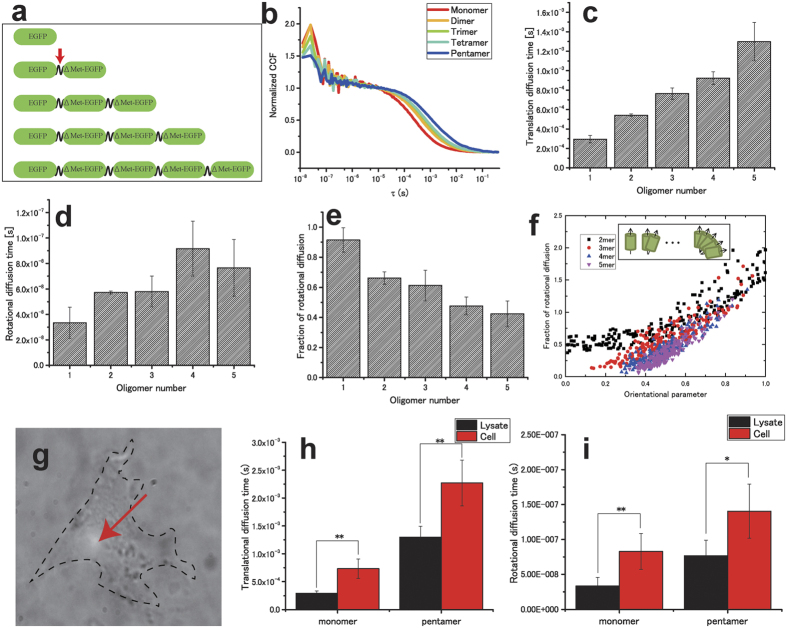
Rotational diffusion of EGFP tandem-oligomers. (**a**) Schematic sequences of the flexible-linker linked EGFP tandem-oligomer. The red arrow shows the flexible linker. Δ Met means the sequence with a lack of initial methionine. (**b**) The typical normalized CCFs of EGFP tandem-oligomers. Optical condition is X-XX. (**c–e**) Translational diffusion time, rotational diffusion time, and fraction of rotational diffusion of EGFP tandem-oligomers, respectively. (**f**) Result of *in silico* simulations of the correlation functions of EGFP tandem-oligomers. The inset figure indicates schematic models of EGFPs. Their transition dipole moments are indicated using black arrows. (**g**) Typical image of the COS-7 cell expressing monomer-EGFP. (**h,i**) Comparison between a cell and a lysate in terms of the translational diffusion time, rotational diffusion time, and fraction of rotational diffusion. The light green spots show the focal points of the pol-FCS measurements. All measurements were performed in X-XX. *p < 0.05, **p < 0.005 (Student’s t-test values).

The EGFP tandem-oligomers were measured to investigate the effect of changing the molecular size in rotational diffusion. If the molecular shape can be assumed as spherical, the change of rotational diffusion coefficient (∝*r*^−3^) has a higher sensitivity to the change of their hydrodynamic radius than that of translational diffusion coefficient (∝*r*^−1^) (Equations 6 and 7). The plasmids of EGFP tandem-oligomers^19^,^20^ were modified in a flexible linker (FL) with a lack of initial methionine in the sequence of EGFPs (ΔMet), and they were used to confirm this theory (Fig. 2a). Typical results of normalized CCF in X-XX are shown in Fig. 2b. Clear CCFs were observed in all cases. The fitted translational diffusion time, rotational diffusion time, and fraction of rotational diffusion are shown in Fig. 2c–e. Each triplet time was fixed to the measured value (Supplementary Fig. 6). There may be circumstances where the EGFPs in tandem-oligomers were not fluorescent by intermolecular quenching or misfolding. For this reason, the molecular brightness did not linearly increase with the increase of the EGFPs size (Supplementary Fig. 7). However, in the FCS case, single tandem-oligomer acts as the only single molecule; thus, these effects should not affect the results of translational and rotational diffusion times. Translational diffusion time gradually increased with molecular size (Fig. 2c). On the other hand, rotational diffusion time did not show this clear tendency (Fig. 2d). This result implies that the shape of the EGFP oligomers was not spherical. In Fig. 2e, the fraction of rotational diffusion also showed dependence on the oligomer size. A greater oligomer number of EGFPs (4 or 5-mer) shows a lower fraction of rotational diffusion when compared to a smaller number (1 or 2-mer). To investigate the orientation of fluorophores in multimeric fluorescent molecules, here we define the orientational parameter OP as:


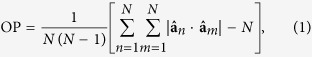


where 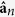 is the unit vector in the transition dipole direction of the n-th fluorophore in the multimer. The orientational parameter represents the degree of anisotropy in the transition dipoles; it will be equal to 1 if all dipoles in the fluorescent multimer are oriented in the same direction. On the other hand, the parameter will be equal to 0 if all the dipole combinations are orthogonal to each other.

Figure 2f shows the orientational parameter dependence on the fraction of rotational diffusion obtained by Monte Carlo simulations. Details of the numerical simulations are described in Supplementary Note 3. The fraction of rotational diffusion in the pol-FCS measurements of multimeric fluorescent molecules positively correlates with the orientation of fluorophores. This is observed because the fractions of rotational diffusion decreased when the number of fluorophores in the multimer was increased, as shown in Fig. 2e. This can be attributed to the decrease of orientation when the number of fluorophores is increased as shown in Fig. 2f (inset). Moreover, the intermolecular quenching and misfolding of the EGFPs may increase the fraction of rotational diffusion.

To demonstrate that the system allows the measurement of rotational diffusion in a cell, pol-FCS measurements were performed in a living cell. Culture cells (COS-7) expressing monomer EGFPs and pentamer EGFPs were prepared (Fig. 2g); then, pol-FCS measurements (X-XX) were performed using them. Figure 2h,i indicate that the translational and rotational diffusion times are longer than those for the cell lysate. In the case of cells, the same tendency of increase in rotational and translational diffusion times is shown as for lysate. *f*_*R*_ increased at pentamer EGFP in cytoplasm (Supplementary Fig. 8); however, it was not observed in the monomer EGFP case.

## Conclusion

In solution measurements of EGFPs, molecular rotational diffusion was clearly observed and it slowed as the viscosity was increased by adding glycerol. From this result, the molecular hydrodynamic radius of EGFP was accurately estimated using the Einstein-Stokes equation. Furthermore, the molecular size-dependency of the EGFP tandem-oligomer translational and rotational diffusion was simultaneously observed using pol-FCS.

Our experimental and simulation results show that the fraction of rotational diffusion reflects the orientation degree of multimeric fluorophores. This orientational information of fluorophore is not easily detected by any other method without fluorescence. It is one of the advantages of the pol-FCS method. We expect that pol-FCS can be applied to study the orientation of multimerized or aggregate-prone proteins such as amyloid beta, which is related to Alzheimer’s disease. In its initial aggregation process, it was pointed out that the orientation of ordered aggregates presumably has an important role in their toxicity and dynamics^21^. However, there are no clear evidences of proofing this concept in living cells. The pol-FCS method can be easily applied to study this.

In cell measurements, translational and rotational diffusion became slower than those in free solutions with the same ratio. If the cellular cytoskeleton is affecting the diffusion, then translational diffusion should become much slower when compared to rotational diffusion. Our results indicate that the EGFPs molecules were freely diffusing in COS-7 cells and the cytoskeleton structure was larger than the observation volume of pol-FCS. Thus, this method may be appropriate to measure tiny areas such as the nanoenvironment of cells. In COS-7, the viscosities of nano- and microenvironments were the same. However, the inner structure (or nanoenvironment) of the cell would be different according to the cell line, cell type, and/or cell cycle. A detailed analysis of changing the inner environment, such as molecular crowding in cell development, will be addressed in a future study. Previous studies indicate that there are some factors in FCS results due to cellular anomalous diffusion in conventional systems^22^,^23^. Nevertheless, pol-FCS is able to clarify the properties of anomalous diffusion in nano- and microenvironment within the tiny observation area.

Compared to time-resolved fluorescent anisotropy decay methods and single molecule tracking methods, pol-FCS is relatively convenient and suitable for living cells. It might open a new horizon in the fields of rotational dynamics of biomolecules in solutions and cells.

## Method

### EGFP purification from *Escherichia coli*

An *Escherichia coli* BL21 (DE3) was transformed using the plasmid encoded his-tag and EGFPs (pRSET-EGFP). Transformant BL21 was cultured by shaking at a temperature of 27 °C for 20 h with 0.5 mM IPTG. The lysate was extracted using sonication in ice, and recovered using centrifugation at 4 °C. Subsequently, the lysate was purified using HPLC (AKTA, GE Healthcare, USA) with a Ni affinity column (HisTrap HP, GE Healthcare, USA). Concentration of purified EGFPs was quantified by FCS using ConfoCor 3. Purified EGFPs were diluted in the PBS buffer: 170 mM NaCl, 27 mM KCl, 81 mM Na_2_HPO_4_, 14.7 mM KH_2_PO_4_.

### Plasmid construction

The plasmids encoded FL-linked EGFP tandem-oligomers (Fig. 2a) for expression in mammalian cells and were constructed as follows. First, an EGFP dimer linked using an FL (EGFP-FL-ΔMet-EGFP) was constructed by inserting PCR products coding FL-ΔMet-EGFP (FL-ΔMet-EGFP) into a modified EGFP-C1 vector. ΔMet-EGFP indicates that the first methionine of EGFPs is removed. In this step, the length of the FL was 27 amino acids, and its sequence was GGGGGSGGGGGSGGGGGSGGGGSGGGS. Subsequently, an EGFP trimer with FLs (EGFP-FL-ΔMet-EGFP-FL-ΔMet-EGFP) was constructed by inserting a part of FL-ΔMet-EGFP into EGFP-FL-ΔMet-EGFP. By repeating this insertion, an EGFP tetramer and pentamer with FLs were constructed. These constructed plasmids contained a multi-cloning site to insert the interested protein into the C-terminal of EGFP oligomers.

### Transfection and preparation of the lysate of mammalian cells

COS-7 cells (5 × 10^5^ cells) were transfected using 1.0 μg plasmids EGFP-C1 and FL-linked EGFP tandem-oligomers (2–5 mer) through Lipofectamine 2000 (Life technologies, USA). The cells were incubated at a temperature of 37 °C with 5% CO_2_. After transfection for 24 h, the cells were scraped and collected into microtubes using centrifugation. Cells were lysed in CelLytic M cell lysis reagent (Sigma-Aldrich, USA). Cell lysates were recovered using centrifugation at two times (3,300× g for 15 min and 90,000× g for 15 min) at a temperature of 4 °C. The concentration of EGFP oligomers in the lysates was determined using FCS measurements, using a 10-time dilution through CelLytic M cell lysis reagent.

### Nonlinear curve fitting

The theoretical equation was fitted to the obtained correlation functions using the nonlinear curve fitting function of OriginPro 2015 (OriginLab Corp., USA).

### Experimental setup

Experimental setup of the pol-FCS system is shown in Fig. 1a. The *z*-axis represents the optical axis and its perpendicular plane is the *x-y* plane. An Ar^+^ laser (IMA101020BOS, Melles Griot, USA) with a wavelength of 488 nm was used as the excitation light for the EGFPs and EGFP tandem-oligomers. The laser light collimated and polarized in the *x*-direction using a polarizer was guided to the light source port for epi-illumination of a fluorescence microscope (Axioplan2, Carl Zeiss, Germany). The guided excitation light was reflected by a dichroic mirror (FT510, Carl Zeiss, Germany) towards the fluorescent solution samples and focused by an objective lens (C-Apochromat 40x, numerical aperture = 1.2, water immersion, Carl Zeiss, Germany). The laser power was 11 μW in the focal plane of the objective lens. Emitted fluorescent light was collected using the same objective lens and passed through an emission filter (BLP01-488R-25, Semrock, USA) and an analyzer. Fluorescent light was divided into two directions using a beam splitter. Fluorescent light was coupled with multimode optical fibers (with a core diameter of 62.5 μm), and its end face worked as a pinhole for the confocal detection of fluorescent light. The two autocorrelation functions (ACFs) and the CCF were calculated from two fluorescence signals detected through APD1 and APD2 (SPCM-CD3017, PerkinElmer, USA) using a digital correlator (ALV-5000E/FAST, ALV-GmbH, Germany). The measurement with a duration of 30 s was repeated 10 times. The averaged auto- and CCFs were obtained. Initially, the calibration of the measurement system was performed using rhodamine 6G as a standard fluorophore (Supplementary Fig. 1).

In the pol-FCS system, four different measurement configurations of polarization can be obtained, as represented by the three letters (X, Y, N) shown in Table 1. For example, “X-XX” and “X-NN.” X, Y, and N means polarization in the X-direction, polarization in the Y-direction, and not polarized, respectively. The first letter of the polarization configuration indicates polarization direction of the excitation laser. The second and third letters of the polarization state means polarization directions of the fluorescent light detected using APD1 (CH. 0) and APD2 (CH. 1), respectively. The CCF of the fluorescence intensities of signals detected using APD1 and APD2 *G*_1,2_(*τ*) is derived as follows:


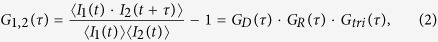


where *τ* is the delay time and *I*_1_, *I*_2_ are the fluorescent intensities detected using APD1 and APD2, respectively. *G*_*D*_, *G*_*R*_, and *G*_*tri*_ are the ACFs of translational diffusion, rotational diffusion, and triplet state relaxation, respectively (see Supplementary Fig. 2):


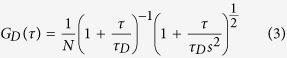



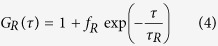



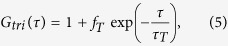


where, *N* is the average number of molecules in the observation volume. *τ*_*D*_ is the relaxation time of translational diffusion. *s* is the structural parameter defined as the ratio of the radii of the longer axis to the shorter axis of the observation volume of this experimental setup. *f*_R_ and *f*_tri_ are the fractions of rotational diffusion and triplet state molecules with respect to the amplitude of the correlation function for translational diffusion *G*_D_(*τ*), respectively. *τ*_R_ and *τ*_T_ means the relaxation time of rotational diffusion and triplet state, respectively.

### Hydrodynamic radius estimation

From the theory of FCS, the translational and rotational diffusion coefficients can be calculated as^5^


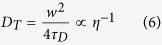



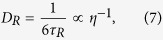


where *w* is the short (lateral) axis radius of the confocal region. Equation 7 shows that the rotational diffusion coefficient is directly obtained from the rotational diffusion time without the use of information about the measurement volume such as *w*. If the molecular shape is spherical, the following Einstein–Stokes equations should be satisfied:


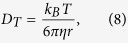



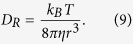


From Eqs 8 and 9, the hydrodynamic radius can be obtained as


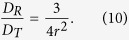


### Measurement in cells

Transiently transfected cells were prepared in 3.5-cm diameter glass bottom dishes (IWAKI, Japan). After transfection for 24 h, the medium was changed from a dulbecco modified eagle medium (Sigma-Aldrich, USA) to Opti-MEM (Invitrogen Life Technologies, Inc., Carlsbad, CA, USA). The dishes were covered using parafilm (Bemis Flexible Packaging, USA) to prevent medium leakage. The observation was performed using the inverted dish. A Xe lamp (Asahi spectra, Japan) was used as a light source to transmit light to the cells.

## Additional Information

**How to cite this article**: Oura, M. *et al*. Polarization-dependent fluorescence correlation spectroscopy for studying structural properties of proteins in living cell. *Sci. Rep.* **6**, 31091; doi: 10.1038/srep31091 (2016).

## Supplementary Material

Supplementary Information

## Figures and Tables

**Table 1:** Polarization configuration of pol-FCS.

Optical condition	Polarizer^a^	Analyzer^b^	Beam Splitter^c^
X-XX	X	X	NPBS
X-YY	X	Y	NPBS
X-NN	X	Not used	NPBS
X-XY	X	Not used	PBS

^a,b^Directions of the polarizer and analyzer of the optical system. ^c^Used beam splitter: nonpolarizing beam splitter (NPBS) or polarizing beam splitter (PBS). Split ratio of the NPBS was 50:50 regardless of the fluorescent light polarization. X and Y means the direction of each optical element: X-direction and Y-direction. N means nonpolarized. Thus, under X-NN condition, fluorescence was corrected as 50:50 regardless of the fluorophore polarization.

## References

[b1] Elson E. & Madge D. Fluorescence correlation spectroscopy: conceptual basis and theory. Biopolymers 13, 1–27 (1974). 10.1002/bip.1974.360130103 4818131

[b2] Rigler R. & Elson E. eds. Fluorescence correlation spectroscopy: theory and applications. Springer, New York (2001).

[b3] Kinjo M. & Rigler R. Ultrasensitive hybridization analysis using fluorescence correlation spectroscopy. Nucl. Acid Res. 23(10) 1795–1799 (1995). 10.1093/nar/23.10.1795 PMC306938 7784185

[b4] Aragon S. & Pecora R. Fluorescence correlation spectroscopy as a probe of molecular dynamics. J. Chem. Phys. 64(4) 1791–1803 (1976).

[b5] Ehrenberg M. & Rigler R. Rotational Brownian motion and fluorescence intensity fluctuations. Chem. Phys. 4, 390–401 (1974).

[b6] Tsay J., Doose S. & Weiss S. Rotational and Translational Diffusion of Peptide-Coated CdSe/CdS/ZnS Nanorods Studied by Fluorescence Correlation Spectroscopy. J. Am. Chem. Soc. 128(5) 1639–1647 (2006). 16448137 10.1021/ja056162i PMC2535805

[b7] Yamamoto J. . Rotational diffusion measurements using polarization-dependent fluorescence correlation spectroscopy based on superconducting nanowire single-photon detector. Opt. Exp. 23(25) 32633–32642 (2015). 10.1364/OE.23.032633 26699052

[b8] Alam S. & Mukhopadhyay A. Translational Anisotropy and Rotational Diffusion of Gold Nanorods in Colloidal Sphere Solutions. Langmuir 31(32) 8780–8785 (2015). 26211935 10.1021/acs.langmuir.5b01682

[b9] Zhang B. . Sensitive Single Particle Method for Characterizing Rapid Rotational and Translational Diffusion and Aspect Ratio of Anisotropic Nanoparticles and Its Application in Immunoassays. Anal. Chem. 85(20) 9433–9438 (2013). 24059451 10.1021/ac4023956

[b10] Wang D. S., Wei S. C., Liao S. C. & Lin C. W. Gold nanorods as probes in two-photon fluorescence correlation spectroscopy: Emerging applications and potential artifacts. Microscopy Res. Tech. 76(9) 882–889 (2013). 10.1002/jemt.22242 23749499

[b11] Barcellona M. . Polarized fluorescence correlation spectroscopy of DNA-DAPI complexes. Microscopy Res. Tech. 65(4–5) 205–217 (2004). 10.1002/jemt.20121 15630690

[b12] Dorfschmid M., Müllen K., Zumbusch A. & Wöll D. Translational and Rotational Diffusion during Radical Bulk Polymerization: A Comparative Investigation by Full Correlation Fluorescence Correlation Spectroscopy (fcFCS). Macromolecules 43(14) 6174–6179 (2010).

[b13] Lee J. . Analysis of quantum rod diffusion by polarized fluorescence correlation spectroscopy. J. Fluoresc. 24(5) 1371–1378 (2014). 24989149 10.1007/s10895-014-1367-2

[b14] Tjandra N., Feller S., Pastor R. & Bax A. Rotational diffusion anisotropy of human ubiquitin from 15N NMR relaxation. J. Am. Chem. Soc. 117(50) 12562–12566 (1995).

[b15] Uji-i H. . Visualizing spatial and temporal heterogeneity of single molecule rotational diffusion in a glassy polymer by defocused wide-field imaging. Polymer 47(7) 2511–2518 (2006).

[b16] Spencer R. & Weber G. Influence of Brownian Rotations and Energy Transfer upon the Measurements of Fluorescence Lifetime. J. Chem. Phys. 52, 1654–1663 (1970).

[b17] Tang J. . Fluorescent protein in inertially injected aqueous nanodroplets. Langmuir 24(9) 4975–4928 (2008). 18366235 10.1021/la800329k

[b18] Yang F., Moss L. & Phillips G. Jr. The molecular structure of green fluorescent protein. Nat. Biochem. 14, 1246–1251 (1996). 10.1038/nbt1096-1246 9631087

[b19] Pack C. G., Saito K., Tamura M. & Kinjo M. Microenvironment and Effect of Energy Depletion in the Nucleus Analyzed by Mobility of Multiple Oligomeric EGFPs. Biophys. J. 91(10) 3921–3936 (2006). 16950841 10.1529/biophysj.105.079467 PMC1630477

[b20] Hendrix J., Schrimpf W., Höller M. & Lamb C. Pulsed Interleaved Excitation Fluctuation Imaging. Biophys. J. 105(4) 848–861 (2013). 23972837 10.1016/j.bpj.2013.05.059 PMC4100079

[b21] Soto C. Unfolding the Role of Protein Misfolding in Neurodegenerative Diseases. Nat. Rev. Neurosci. 4(1) 49–60 (2003). 12511861 10.1038/nrn1007

[b22] Egelman E. “Comprehensive Biophysics, 1st edition” Academic Press, “2-11-4-5 Anomalous diffusion” (2012).

[b23] Zorrilla S., Hink M. A., Visser A. J. & Lillo M. P. Translational and rotational motions of protein in a protein crowded environment. Biophysical Chemistry 125, 298–305 (2007). 17007994 10.1016/j.bpc.2006.09.003

