# Supplementary material for: Polarization-dependent fluorescence correlation spectroscopy for studying structural properties of proteins in living cell
Source: Sci Rep. 2016 Aug 4;6:31091. doi: 10.1038/srep31091 (PMC4973283; doi:10.1038/srep31091)
Supplement: Supplementary Information [file srep31091-s1.pdf]

Supplementary information

Submitted to: Scientific reports

Polarization-dependent fluorescence correlation spectroscopy for studying structural properties of proteins in living cell.

Makoto Oura[1,*], Johtaro Yamamoto[2,*], Hideto Ishikawa[1], Shintaro Mikuni[2], Ryousuke Fukushima[1], and Masataka Kinjo[2]

[1] Laboratory of Molecular Cell Dynamics, Graduate School of Life Science, Hokkaido University, Sapporo, 001-0021, Japan

[2] Laboratory of Molecular Cell Dynamics, Faculty of Advanced Life Science, Hokkaido University, Sapporo, 001-0021, Japan

(* equal contribution)

Supplementary Note 1, Optical calibration method. To calibrate an optical system for the pol-FCS system, rhodamine 6G (Rh6G), which is widely used as a standard fluorescent molecule for FCS measurements, was measured. The autocorrelation function (ACF) and the CCFs of the Rh6G under X-NN optical condition are shown in **Supplementary Fig. 1**. In the case of ACF, it was observed a large fraction of after-pulsing in the microsecond region of the correlation functions (**Supplementary Fig. 1b**: red arrow) and a decrease of the correlation functions in the nanosecond region (**Supplementary Fig. 1b**: green arrow). However, those were not in the CCFs because the after-pulsing, detector dead-time, and specific noise independently occurred in each APD. This result indicates that these fractions caused by the detector noise can be removed from the correlation functions in the range from nanoseconds to microseconds because the detector dead-time and after-pulsing fraction of ACF were canceled via CCFs calculation. Nonlinear curve fitting to CCF without the rotational diffusion component was performed because this experiment aimed to determine the character of the optical system from the shape of the CCF affected by translational diffusion. In this fitting, *s* was 5 to obtain the reliable value of the parameter. However, when s was fixed, the standard residuals were tiny. Therefore, *s* value was believed to be a suitable value for these experiments.

Supplementary Note 2, Estimation method of triplet time. Triplet time was obtained using **Eq. S1** and was found to be 1.24 μs, which was measured using ConfoCor 3 (**Supplementary Fig. 3**). In terms of this quantitative analysis, translational and rotational diffusion times clearly decreased as the concentration of glycerol was increased. Furthermore, the fraction of rotational diffusion was changed. The difference between the results of experiments without glycerol and those using glycerol is significant (among the series with glycerol were observed. It means that some changes are related to the glycerol presence, and the results are independent on the solution viscosity.

$$G(\tau) = \left[\frac{1 - f_T + f_T \exp(-\frac{\tau}{\tau_T})}{N(1 - f_T)} \right] \left[\frac{1}{1 + (\frac{\tau}{\tau_D})} \right] \left[\frac{1}{1 + \left(\frac{1}{s}\right)^2 \left(\frac{\tau}{\tau_D}\right)} \right]^{\frac{1}{2}} \quad \text{(S1)}$$

Supplementary Note 3, Numerical simulation of the fraction of rotational diffusion in pol-FCS using the Monte Carlo method.

Fractions of rotational diffusion were numerically simulated for EGFP oligomers using the Monte Carlo method. Here, we assumed for simplicity that the transition dipole directions of absorption

and emission of EGFPs were the same, and the fluorescence lifetime is much faster than the rotational diffusion time. Furthermore, we assumed that the fluorescence quantum yield of the EGFPs and the quantum efficiency of the photon detector are 1.0

The probability that fluorophore absorbs a photon can be expressed using the normalized transition moment of absorption and emission of the n-th EGFP in the EGFP oligomer $\hat{\boldsymbol{\mu}}_n$ [1,2]:

$$p_{\mathrm{a}}\left(\hat{\boldsymbol{\mu}}_n\right) = 3\left(\hat{\boldsymbol{\mu}}_n \cdot \hat{\mathbf{e}}_{\mathrm{p}}\right)^2, \tag{S2}$$

where $\hat{\mathbf{e}}_{\mathrm{p}}$ is the unit vector with the polarization direction of the excitation laser (the polarizer direction). Similarly, the probability that a photon emitted by an excited EGFP can be detected is as follows:

$$p_{\mathrm{e}}\left(\hat{\boldsymbol{\mu}}_n\right) = 3\left(\hat{\boldsymbol{\mu}}_n \cdot \hat{\mathbf{e}}_{\mathrm{a}}\right)^2, \tag{S3}$$

where $\hat{\mathbf{e}}_{\mathrm{a}}$ is the unit vector with the polarization direction of the detected fluorescent light (the analyzer direction). In that case, the detected fluorescence intensity using the detector is given by

$$I_{\mathrm{single}}\left(\hat{\boldsymbol{\mu}}_n\right) = 9I_{\mathrm{ex}} \cdot p_{\mathrm{a}}\left(\hat{\boldsymbol{\mu}}_n\right) \cdot p_{\mathrm{e}}\left(\hat{\boldsymbol{\mu}}_n\right) \tag{S4}$$

where I_{ex} is the intensity of the excitation laser at the EGFPs position.

Now we consider that the N_{o} -mer EGFP (EGFP oligomer) rotates randomly: namely, rotational diffusion, and the EGFP oligomers do not diffuse translationally. The fluorescence intensity emitted by an EGFP oligomer is detected M times using the pol-FCS setup. Fluorescence signal is then given by

$$I_m = \sum_{n=1}^{N_{\mathrm{o}}} I_{\mathrm{single}}\left(\hat{\boldsymbol{\mu}}_{n,m}\right), \tag{S5}$$

where $\hat{\boldsymbol{\mu}}_{n,m}$ is the transition moment of the n-th EGFP included at an EGFP oligomer at the m-th detection of the fluorescent light. The transition moment of a randomly rotating EGFP oligomer was expressed as follows:

$$\hat{\boldsymbol{\mu}}_{n,m} = \mathbf{R}_m^x\left(\theta_m\right) \cdot \mathbf{R}_m^y\left(\phi_m\right) \cdot \mathbf{R}_m^z\left(\rho_m\right) \cdot \hat{\boldsymbol{\mu}}_{n,m-1}, \tag{S6}$$

where $\mathbf{R}_m^x$, $\mathbf{R}_m^y$, and $\mathbf{R}_m^z$ are the rotational matrices of x, y, and z axes, respectively. θ, ϕ,

and ρ are the rotation angles, and those were randomly defined in the range of 0 to 2π. An EGFP oligomer rotates maintaining the orientation among all the EGFPs included by the EGFP oligomer.

The auto-correlation function of the fluorescence intensity signal is given by:

$$G(\tau)=\frac{\langle I(t)\cdot I(t+\tau)\rangle}{\langle I(t)\rangle^2}=\frac{\langle \delta I(t)\cdot \delta I(t+\tau)\rangle}{\langle I(t)\rangle^2}+1$$

$$=f_R\exp\left(-\frac{\tau}{\tau_R}\right)+1 \qquad \text{(S7)}$$

where f_R and τ_R are the fractions of rotational diffusion and rotational diffusion time. The fraction of rotational diffusion can be expressed by

$$f_R=G(0)-1=\frac{\sigma^2}{\bar{I}^2}, \qquad \text{(S8)}$$

where

$$\sigma^2=\frac{1}{M}\sum_{m=1}^{M}\left(I_m-\bar{I}\right)^2, \qquad \text{(S9)}$$

$$\bar{I}^2=\frac{1}{M}\sum_{m=1}^{M}I_m. \qquad \text{(S10)}$$

In the numerical simulations, initial orientations of each EGFP included in the EGFP oligomer $\hat{\boldsymbol{\mu}}_{n,0}$ were randomly defined. Next, the fluorescence intensity signal was generated using **Eqs. S5 and S6**. Finally, the fraction of rotational diffusion was obtained using **Eq. S8**.

References

1. M. Ehrenberg and R. Rigler, “Rotational Brownian motion and fluorescence intensity fluctuations,” *Chem. Phys.* **4** (1974) 390-401.
2. P. Kask, P. Piksarv, M. Pooga, Ü. Mets, and E. Lippmaa, “Separation of the rotational contribution in fluorescence correlation experiments,” *Biophys. J.* **55** (1989) 213-220.

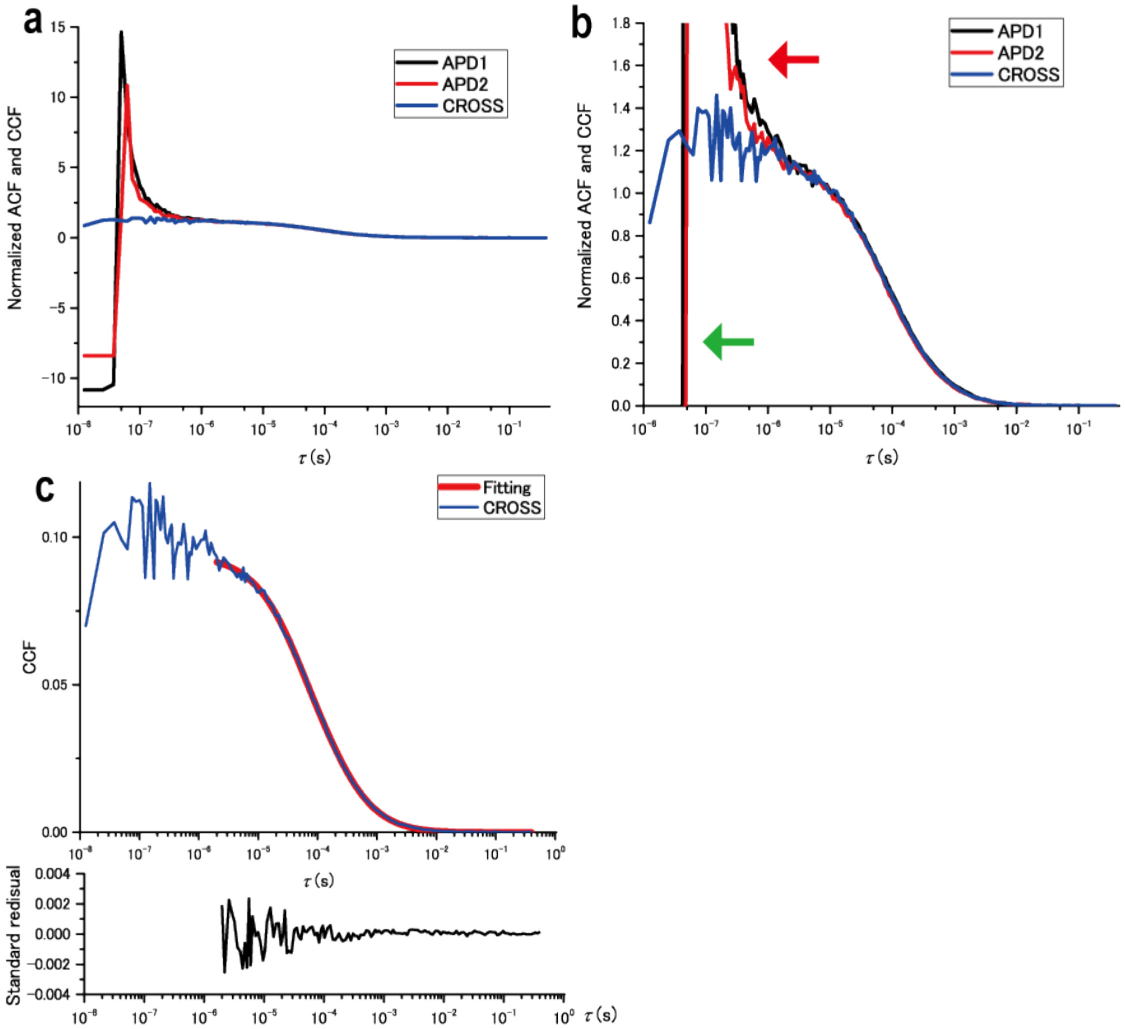

Supplementary Figure 1, Experimental calibration of the setup. (**a**) Normalized ACF and CCF of rhodamine 6G. (**b**) Enlarged graph of (**a**). Red and green arrows indicate after-pulsing and dead-time fraction, respectively. (**c**) The CCF result and the fitting curve. The standard residual is shown below the graph.

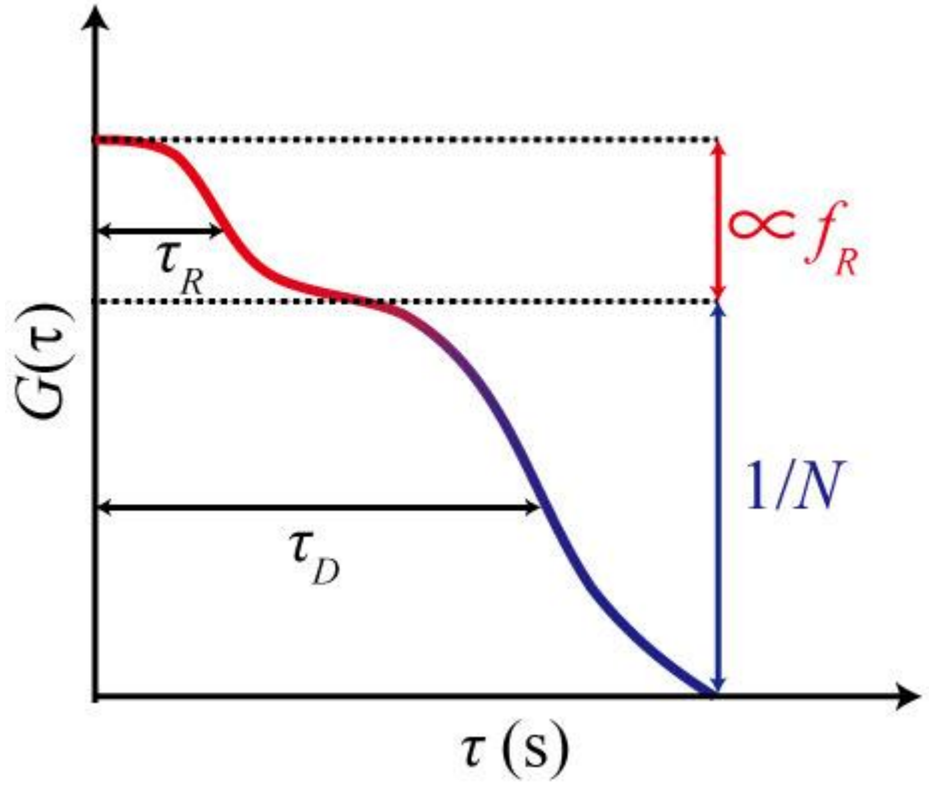

Supplementary Figure 2, Schematic correlation function with fitting parameters.

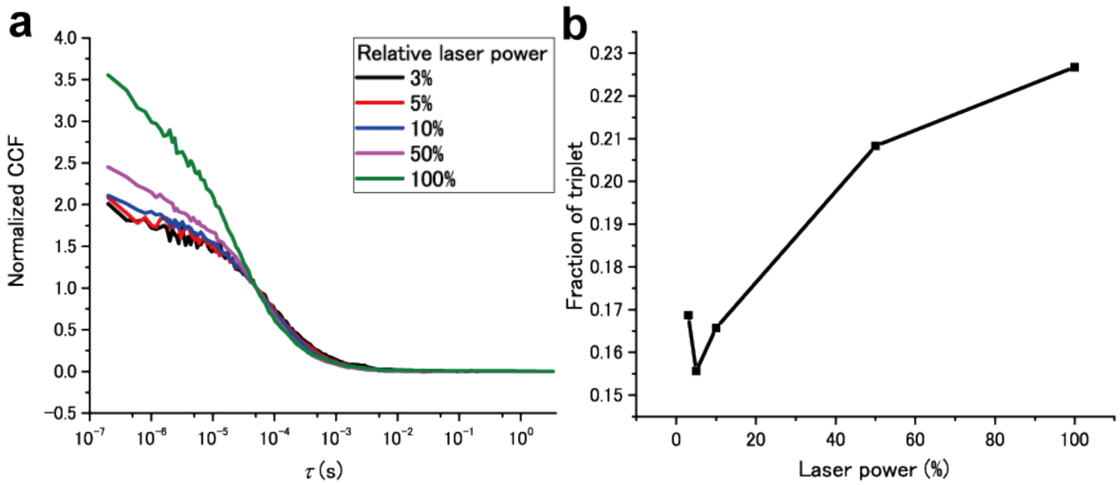

Supplementary Figure 3, Laser power dependence of the triplet state relaxation time of recombinant EGFPs using Confocor3. (**a**) Normalized CCFs of purified recombinant EGFPs. (**b**) Fitted fraction of triplet as a function of the laser power of excitation light.

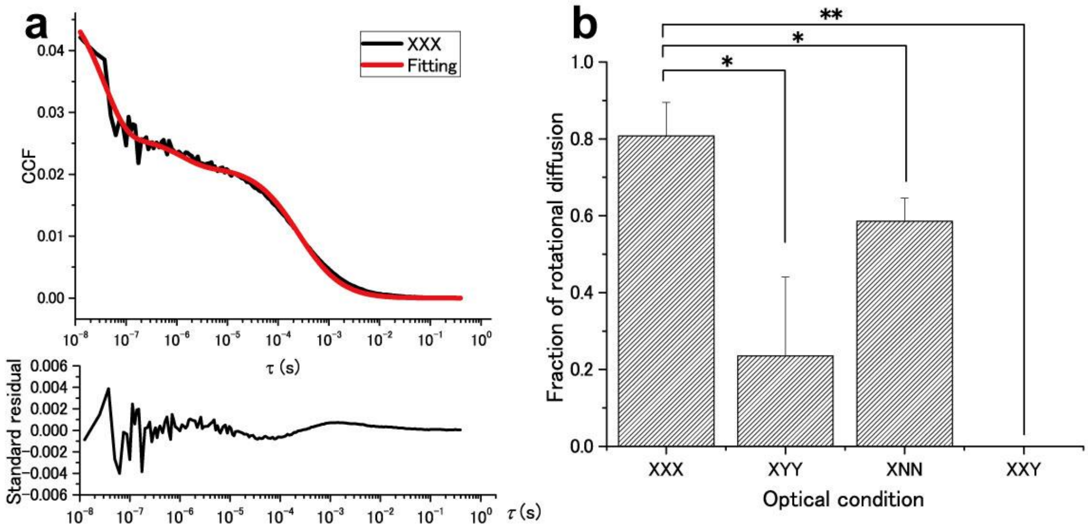

Supplementary Figure 4, Fitting result of the X-XX case using EGFPs. (a) Typical fitting result and standard residual. (b) Summarized fraction of rotational diffusion of each four optical condition. *: $p < 0.05$, **: $p < 0.001$ (Student's t-test values).

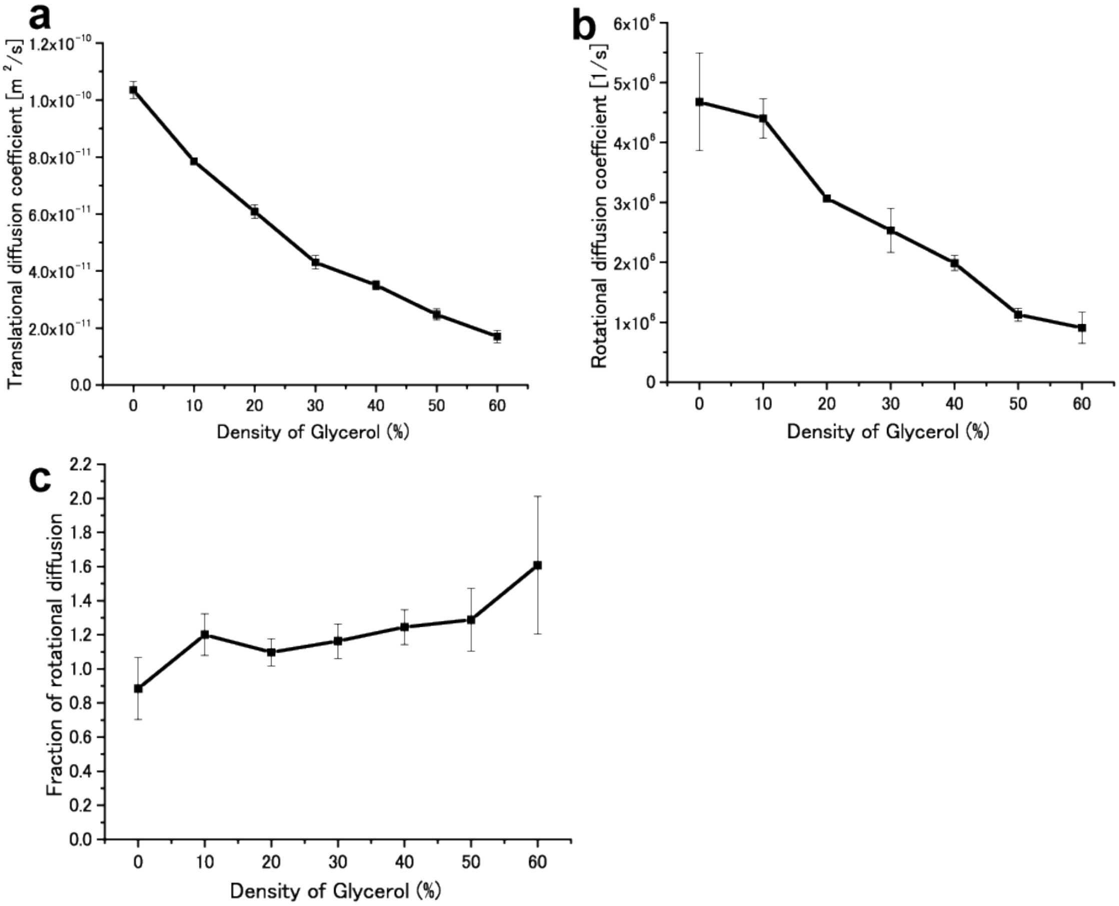

Supplementary Figure 5, Observed diffusion coefficients and the fraction of rotational diffusion of EGFPs diluted in a glycerol and water solution. (**a–c**) The translational diffusion coefficients, rotational diffusion coefficients, and fraction of rotational diffusion.

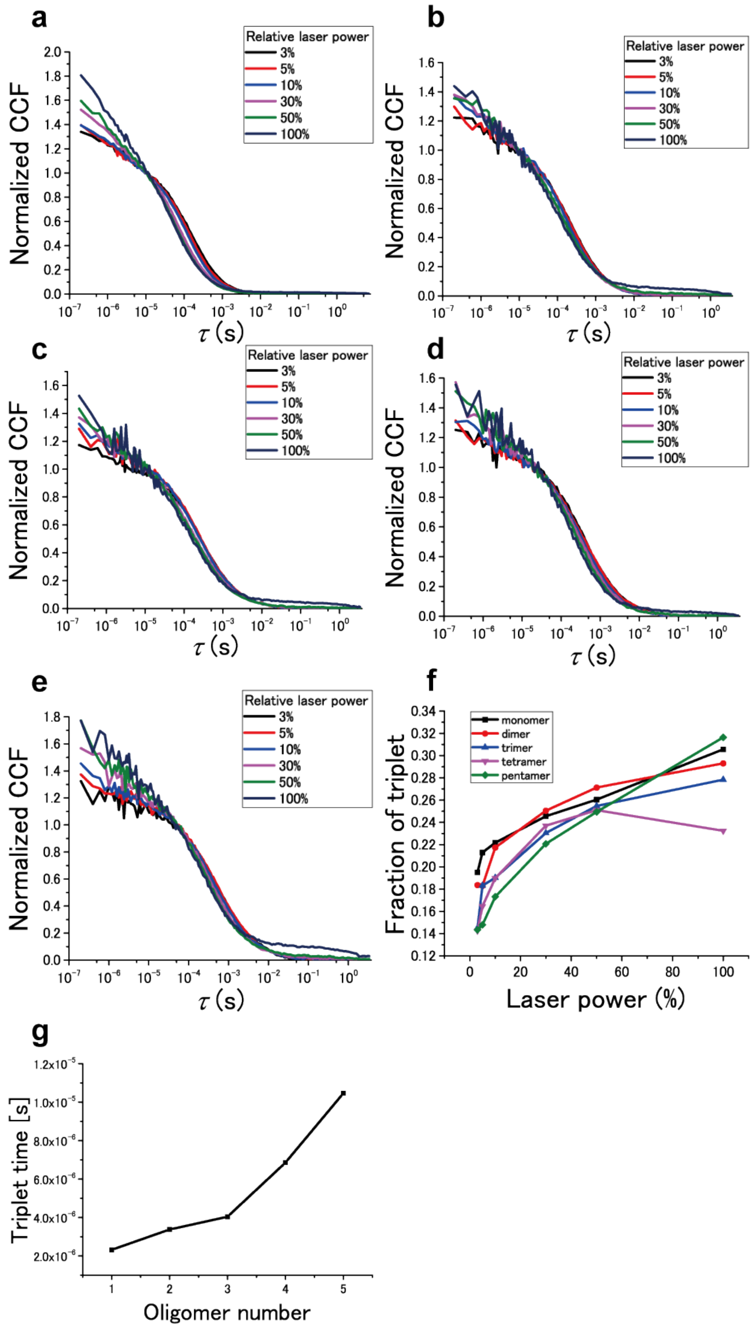

Supplementary Figure 6, Triplet state relaxation time of the multimer EGFPs in cell lysate. (**a**) Normalized CCFs of the monomer EGFPs in cell lysate. (**b**) Normalized CCFs of the dimer EGFPs in cell lysate. (**c**) Normalized CCFs of the trimer EGFPs in cell lysate. (**d**) Normalized CCFs of the tetramer EGFPs in cell lysate. (**e**) Normalized CCFs of the pentamer EGFPs in cell lysate. (**f**) Fitted fraction (amplitude) of the triplet state as a function of the excitation laser power. (**g**) Fitted triplet time as a function of the oligomer number (1–5).

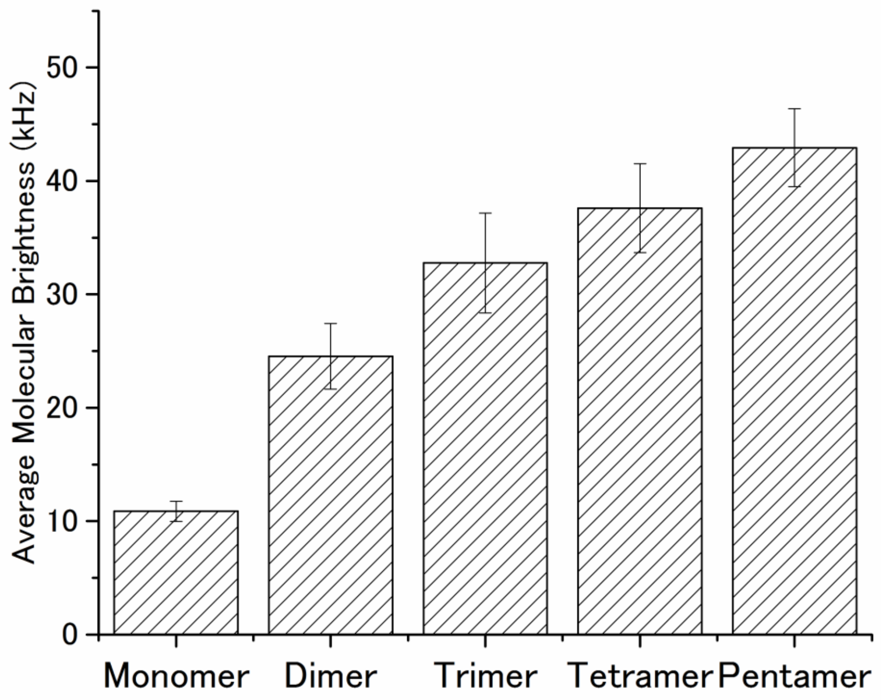

Supplementary Figure 7, Molecular brightness of EGFP tandem-oligomers. Each brightness was measured using a commercial FCS system (Confocor 3, LSM 510). Laser wavelength: 488 nm, Ar+ ion laser. HFT 405/488 and BP505-610 were used. Acquisition time was 10 s looped 10 times. N = 3. This measurement followed the experimental methods used for measuring the EGFP-dimer[3] and oligomers[4]. Molecular brightness of the EGFP tandem-oligomer constructed using multi cloning site was previously performed. This experimental data is in agreement with the previous results.

3. Oasa, S., Sasaki, A., Yamamoto, J., Mikuni, S. & Kinjo, M. Homodimerization of glucocorticoid receptor from single cells investigated using fluorescence correlation spectroscopy and microwells. *FEBS Lett.* **589**(17) 2171-2178 (2015).
4. Hendrix, J., Schrimpf, W., Höller, M. & Lamb, DC. Pulsed interleaved excitation fluctuation imaging. *Biopys. J.* **105**(4) 848-861 (2013).

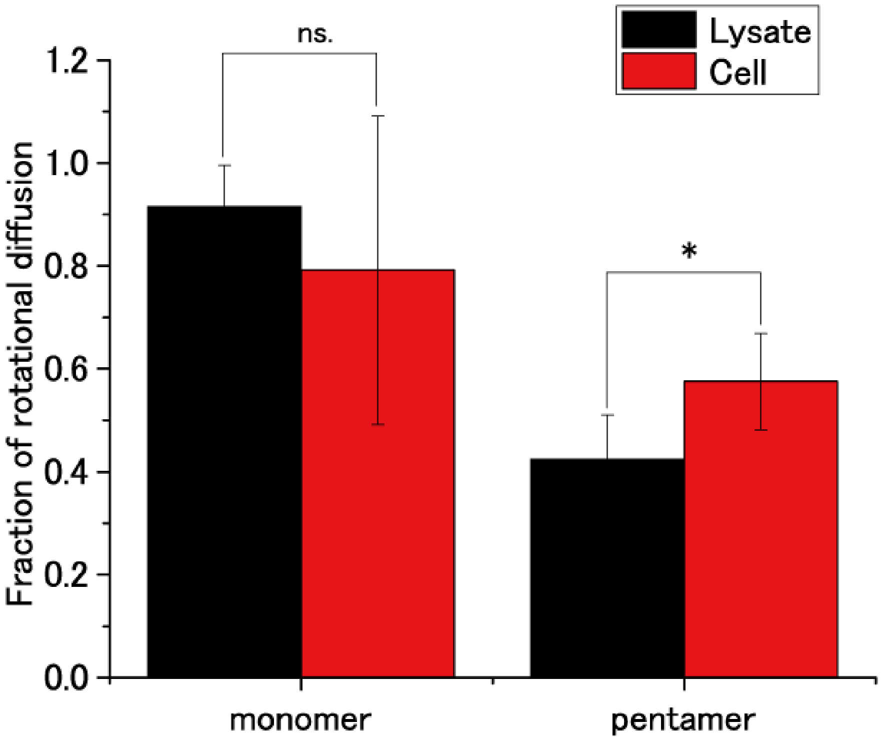

Supplementary Figure 8, Fraction of rotational diffusion in cell lysate and in cell. *: $p < 0.05$ (Student’s t-test values).
